# Inductive interactions mediated by interplay of asymmetric signalling underlie development of adult haematopoietic stem cells

**DOI:** 10.1038/ncomms10784

**Published:** 2016-03-08

**Authors:** Céline Souilhol, Christèle Gonneau, Javier G. Lendinez, Antoniana Batsivari, Stanislav Rybtsov, Heather Wilson, Lucia Morgado-Palacin, David Hills, Samir Taoudi, Jennifer Antonchuk, Suling Zhao, Alexander Medvinsky

**Affiliations:** 1 Institute for Stem Cell Research, Medical Research Council Centre for Regenerative Medicine, University of Edinburgh, SCRM Bioquarter, 5 Little France Drive, Edinburgh EH16 4UU, Scotland, UK; 2 Molecular Medicine Division, The Walter and Eliza Hall Institute of Medical Research, Victoria 3052 Melbourne, Australia; 3 Department of Medical Biology, University of Melbourne, Victoria 3052 Melbourne, Australia; 4 Cancer and Haematology Division, The Walter and Eliza Hall Institute of Medical Research, Victoria 3052 Melbourne, Australia; 5 STEMCELL Technologies Inc., Vancouver, British Columbia V5Z 1B3, Canada

## Abstract

During embryonic development, adult haematopoietic stem cells (HSCs) emerge preferentially in the ventral domain of the aorta in the aorta–gonad–mesonephros (AGM) region. Several signalling pathways such as Notch, Wnt, Shh and RA are implicated in this process, yet how these interact to regulate the emergence of HSCs has not previously been described in mammals. Using a combination of *ex vivo* and *in vivo* approaches, we report here that stage-specific reciprocal dorso–ventral inductive interactions and lateral input from the urogenital ridges are required to drive HSC development in the aorta. Our study strongly suggests that these inductive interactions in the AGM region are mediated by the interplay between spatially polarized signalling pathways. Specifically, Shh produced in the dorsal region of the AGM, stem cell factor in the ventral and lateral regions, and BMP inhibitory signals in the ventral tissue are integral parts of the regulatory system involved in the development of HSCs.

## Abstract (web summary)

It is unclear how the microenvironment of the aorta-gonad-mesonephros influences haematopoietic stem cell (HSC) production early in mouse development. Here, Souilhol *et al.* use an *in vitro* aggregate system as a tool to understand how several pathways, BMP, SCF and Shh, may regulate HSC production.

## Graphical abstract

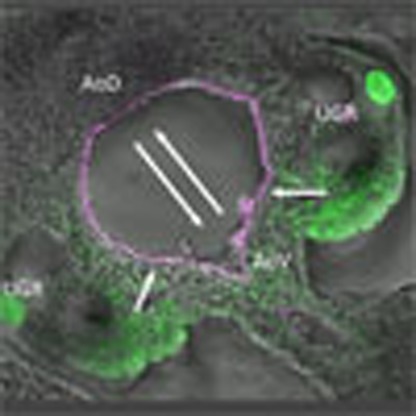

Haematopoietic stem cells (HSCs) lie at the foundation of the adult haematopoietic system, and give rise to cells of all blood lineages throughout the lifespan of an organism. An important property of adult (definitive) haematopoietic stem cells (dHSCs) is that they are capable of long-term reconstitution of the haematopoietic system upon transplantation into irradiated recipients. In the mouse, such cells develop by embryonic stages E10–E11 in the aorta–gonad–mesonephros (AGM) region^1^,^2^,^3^,^4^. An *ex vivo* approach showed that the AGM region has a robust autonomous capacity to generate dHSCs^1^. The AGM region comprises the dorsal aorta flanked on both sides by the urogenital ridges (UGRs), which contain embryonic rudiments of kidney and mesonephros. HSCs develop in a polarized manner, predominantly in the ventral floor of the dorsal aorta (AoV), more rarely in the dorsal domain of the dorsal aorta (AoD), and are absent in the UGRs^2^,^5^,^6^,^7^. Localization of dHSCs to the AoV in mouse and human embryos was shown by long-term reconstitution experiments^5^,^6^.

Abundant evidence indicates that during development, a specialized embryonic endothelial compartment known as haematogenic (or haemogenic) endothelium gives rise to haematopoietic stem and progenitors cells^7^,^8^,^9^,^10^. The haematopoietic programme in various vertebrate models is executed predominantly in the AoV, and is recognized by the expression of essential haematopoietic transcription factors, for example, Runx1 and cKit, and the appearance of clusters of haematopoietic cells budding from the endothelium of the dorsal aorta^6^,^8^,^9^,^11^,^12^,^13^,^14^.

It is broadly accepted that HSCs develop from the haematogenic endothelium within intra-aortic clusters. This transition involves several consecutive maturation steps of HSC precursors: pro-HSCs→pre-HSC type I→pre-HSC type II→dHSC^15^,^16^,^17^. All these precursors express endothelial markers, such as vascular-endothelial cadherin (VC) and CD31, and sequentially upregulate haematopoietic surface markers: CD41 (pro-HSCs), CD43 (pre-HSC type I) and finally CD45 (pre-HSC type II). This maturation process occurs in the dorsal aorta between E9 and E11. Specifically, pro-HSCs emerge at E9, pre-HSCs Type I appear at E10 and pre-HSCs type II predominantly at E11. Unlike dHSCs, pre-HSCs cannot reconstitute the adult haematopoietic system by direct transplantation and require prior maturation in an embryonic or neonatal environment^15^,^16^,^17^,^18^,^19^.

A number of signalling pathways (Notch, Wnt, retinoic acid, interleukin-3 and inflammatory) have been implicated in HSC development; however, a coherent picture is yet to be elucidated^15^,^17^,^20^,^21^,^22^,^23^,^24^,^25^,^26^,^27^,^28^,^29^,^30^,^31^. HSC precursors (pro-HSCs, pre-HSCs type I and pre-HSCs type II) express cKit^17^ from early developmental stages. A recent study has shown that the cKit ligand, known as stem cell factor (SCF), is a key regulator driving maturation of these HSC precursors into dHSCs in the AGM region^17^, which is in agreement with the marked decline of HSC activity in SCF mutant mice^32^,^33^. In the adult, SCF is critically important for HSC maintenance in the bone marrow niche, mainly in the endothelial compartment^32^. Sonic Hedgehog (Shh) and bone morphogenetic protein 4 (BMP4) pathways are also important mediators; in zebrafish, these two morphogenes are involved in arterial specification and haematopoietic patterning, respectively^34^,^35^. In the mouse, subaortic BMP4 and Shh/Indian Hedgehog derived from gut were also proposed to be responsible for HSC development^36^,^37^.

During development, interactions between spatially segregated compartments are essential for tissue patterning and specification, and are often mediated by gradients of secreted molecules^38^,^39^,^40^. Molecules secreted by distant tissues, such as somites, can influence HSC development in the AGM region^41^,^42^,^43^,^44^,^45^. Developing HSCs are embedded in the complex AGM microenvironment, suggesting that HSC development may require signals derived from different compartments of the AGM region. We sought to test this hypothesis. However, the analysis of HSC development *in vivo* is significantly hampered by low accessibility of embryos developing *in utero*, fast maturation of dHSCs, lack of uniquely specific markers for HSC precursors and their low numbers in the AGM region. Therefore, we employed here a robust *ex vivo* culture system that models HSC development in the embryo in combination with functional HSC analysis using *in vivo* long-term reconstitution assay^15^,^16^,^17^. Specifically, to study interactions between AGM subregions, we took advantage of the *in vitro* reaggregation system that enables close juxtaposition of cell types^15^.

We show that interactions between three compartments of the AGM, the AoV, the AoD and the UGRs, are necessary for efficient generation of dHSCs. First, we show that dHSC activity in the isolated E10.5 AoV is limited but can be significantly enhanced by co-culture with the AoD, and that this is mediated at least partly by Shh, secreted dorsally *in vivo*. Second, while HSC activity in isolated E11.5 AoD is limited, co-culture with a competent AoV microenvironment activates dHSC generation in the AoD. We found that this effect is mediated by SCF, which is secreted abundantly by the AoV stroma *in vivo* as shown here. Third, we show that downregulation of BMP4 signalling by BMP antagonist Noggin, which is present at high levels in the AoV and especially in intra-aortic clusters as revealed here by *in vivo* observations, is required for HSC development. Fourth, UGRs, which express high levels of SCF, also enhance HSC development in the dorsal aorta.

Our results based on *in vivo* observations and *ex vivo* modelling strongly suggest that juxtaposed, anatomically distinct domains within the AGM region create a complex landscape of interactive signals that underpins HSC development.

## Results

### Pre-HSCs localize preferentially to the AoV

As dHSCs mature from pre-HSCs, we investigated whether the emergence of dHSC predominantly in the AoV^6^ is a result of asymmetric (ventralized) distribution of pre-HSCs. Dorsal aortae were separated from UGRs and bisected into AoV and AoD (including notochord) as described previously^6^ (Supplementary Fig. 1a). The different domains were then directly transplanted into irradiated mice to detect dHSCs. We first confirmed our previous observation that at E11.5 dHSCs appear almost exclusively in the AoV, although some dHSCs were in the AoD and engrafted few recipients at high level (Supplementary Fig. 1b). Limiting dilution analysis showed that dHSCs are approximately four times more frequent in the AoV compared with AoD. UGRs did not contain HSCs in line with previous reports^2^,^6^.

**Figure 1:**
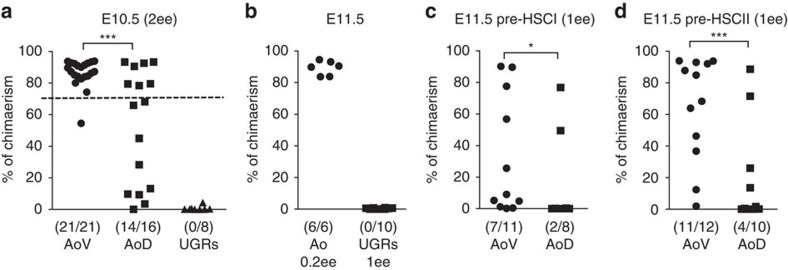
Localization of pre-HSCs in the AGM region. (**a**) E10.5 AoV, AoD and UGRs were co-aggregated with OP9 and cultured for 5 days, and the formation of dHSCs was then tested by transplantation into irradiated mice (2 e.e. per recipient; AoV: six independent experiments; AoD: four independent experiments; UGRs: two independent experiments). Dashed line indicates the cutoff for high-level engraftment (>70% donor chimaerism). (**b**) E11.5 aortas and UGRs were transplanted after reaggregate culture (Ao: 0.2 e.e. per recipient and UGRs: 1 e.e. per recipient; two independent experiments). (**c**,**d**) Pre-HSCs type I (VC^+^CD45^−^) (**c**) or type II (VC^+^CD45^+^) (**d**) sorted from E11.5 AoV and AoD were co-aggregated with OP9 cells and transplanted after culture (1 e.e. per recipient; two independent experiments). (**a**–**d**) Levels of engraftment are plotted, and number of repopulated versus total number of transplanted mice are shown in brackets. Number of embryo equivalents (ee) injected in each experiment are indicated on the graphs. (*P<0.05; ***P<0.005; Mann–Whitney *U*-test). In all these experiments, tissues were cultured with three growth factors (Flt3I, Il3 and SCF). AGM, aorta–gonad–mesonephros region; Ao, dorsal Aorta; AoV, ventral domain of the dorsal aorta; AoD, dorsal domain of the dorsal aorta; UGRs, urogenital ridges.

We then investigated the spatial distribution of pre-HSCs type I and pre-HSCs type II in E10.5–E11.5 embryos using the OP9 co-culture system supplemented with Il3+SCF+Flt3 (termed 3GF), which allows pre-HSCs (which do not engraft by direct transplantation) to mature into dHSC that become detectable by long-term repopulation assay as described previously^16^. Doses of transplanted cells (expressed in embryo equivalents, e.e.) were chosen based on the requirements of individual experiments (explained in Methods section). In these experiments (Fig. 1), the dose injected was high (1–2 e.e.) to detect potentially low dHSC numbers in AoD and UGRs.

We have shown previously that E10.5 AGM region mainly contains type I pre-HSCs, whereas at E11.5, type I and type II pre-HSCs co-exist^16^. Dissected E10.5 AGM regions co-cultured with OP9 in 3GF for 5 days were transplanted into adult irradiated recipients. Out of 21 recipients that received cultured AoV, 20 showed high levels (>70%) of donor-derived long-term haematopoietic chimerism (Fig. 1a). In contrast, only 7 out of 16 recipients of cultured AoD were repopulated at high levels (>70%), while the remaining recipients showed lower or no repopulation (7 and 2, respectively). Cultured UGRs did not produce dHSCs (Fig. 1a). Thus, we conclude that the E10.5 AoD does contain pre-HSCs but at significantly lower numbers than the AoV.

We then investigated whether pre-HSCs localization changes in E11.5 embryos and found that pre-HSCs were still exclusively localized to the dorsal aorta; UGRs carefully separated from the lateral mesenchyme adjacent to the dorsal aorta did not give any repopulation after culture (Fig. 1b). To establish the location of pre-HSCs within the E11.5 dorsal aorta, cell populations enriched for pre-HSCs type I (VC^+^CD45^-^) and pre-HSCs type II (VC^+^CD45^+^) were sorted from AoV and AoD, and co-cultured with OP9 stromal cells in the presence of 3GF as described previously^16^. We again were able to detect pre-HSC activity in AoD although at lower levels than in AoV. After maturation *ex vivo*, pre-HSCs type I from AoV and AoD repopulated 7 of 11 and 2 of 8 recipients, respectively (Fig. 1c). Similarly, cultured pre-HSCs type II from AoV and AoD repopulated 11 out of 12 and 4 out of 10 recipients, respectively (Fig. 1d). In all cases, multilineage engraftment was confirmed (Supplementary Fig. 2). These data show that pre-HSCs are significantly enriched in AoV.

### Reciprocal inductive interactions between AoD and AoV

**Figure 2:**
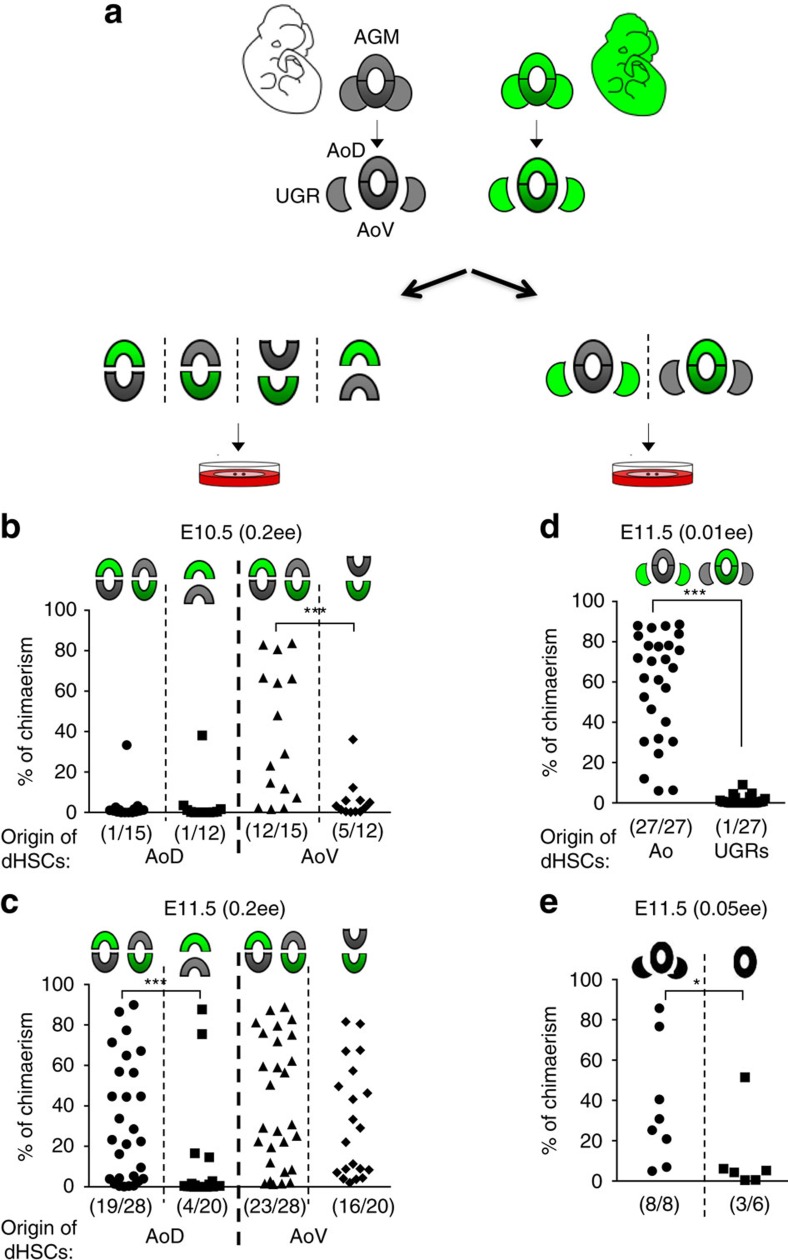
Inductive interactions between AoV, AoD and UGRs as revealed by an *ex vivo* model system. (**a**) Experimental design: the ventral domain (AoV) and the dorsal domain (AoD) of the aorta, and the urogenital ridges (UGRs) from wild-type (WT) and GFP+ embryos were subdissected, and chimeric reaggregates from tissues of these two genotypes were generated. Left column: to test interactions between AoV and AoD, chimeric AoV//AoD reaggregates were generated and transplanted into irradiated recipients after 4–5 days of culture (**b**,**c**). Right column: to test interactions between Ao and UGRs, chimeric Ao//UGR reaggregates were generated and transplanted into irradiated recipients after 4–5 days culture (**d**). GFP+ and/or GFP− donor-derived long-term repopulation allowed us to conclude whether dHSCs originated from AoV, AoD or UGRs. Accordingly, the tissue of origin of donor dHSCs is indicated below each graph. (**b**) E10.5 aortas from WT and GFP embryos were used to generate chimeric reaggregates as depicted schematically above plots. The reciprocal combination of WT and GFP tissues was used to generate AoV//AoD reaggregates. The tissue source of dHSCs is shown separately in the leftmost (AoD) and rightmost (AoV) columns as indicated below the plot (0.2 e.e. per recipient; two independent experiments). (**c**) E11.5 aortas from WT and GFP embryos were used to generate chimeric reaggregates. The tissue source of dHSCs is shown separately in the leftmost (AoD) and rightmost (AoV) columns as indicated below the plot (0.2 e.e. per recipient; two independent experiments). (**d**) E11.5 aortas (Ao) and UGRs from WT and GFP embryos were used to generate Ao//UGR chimeric reaggregates. As depicted schematically above the plot, the reciprocal combination of WT and GFP tissues was used to generate Ao//UGR reaggregates. The tissue source of dHSCs is shown separately in left (Ao) and right (UGRs) columns as indicated below the plot (0.01 e.e. per recipient; six independent experiments). (**e**) Reaggregation of WT Ao with UGRs generate more dHSCs than Ao alone (0.05 e.e. per recipient; two independent experiments). (**b**–**e**) In all these experiments, tissues were cultured with three growth factors.

To explore hypothetical interactions between AoD and AoV, we made use of a dissociation–reaggregation system that recapitulates HSC development *ex vivo*^15^. This system allowed us to integrate AGM domains in a three-dimensional tissue-like organoid^15^ and study their interactions in HSC development. To track the origin of dHSCs, AoV and AoD from wild-type (WT) and green fluorescent protein (GFP) embryos with constitutive expression of GFP^46^ were co-aggregated (termed AoV//AoD co-aggregates) and cultured for 5 days in the presence of 3GF before transplantation (Fig. 2a). Mice transplanted with AoV//AoD co-aggregates can be reconstituted by dHSCs coming from AoD and AoV. The presence of GFP allowed the individual contributions of AoV and AoD to the total repopulation level within the same mouse to be assessed (Fig. 2b,c). This is presented in two separate columns in the graph. Namely, while columns 1 and 3 represent the same recipient mice, the former shows exclusively the contribution of the AoD and the latter shows exclusively the contribution of the AoV into each recipient. To assess the influence of AoD and AoV interaction on HSC development, the repopulation by co-aggregated AoD (column 1) or AoV (column 3) can then be compared with repopulation by independently cultured AoD (column 2) or AoV (column 4). All experiments included reciprocal use of WT and GFP tissues in AoV//AoD co-aggregates, and we observed no difference in repopulation properties between WT and GFP embryos. Homotypic AoV//AoV and AoD//AoD co-aggregates were always used as controls. Note that in these experiments, only 0.2 e.e. were injected per recipient, to ensure that the repopulation levels were not saturated and to allow any inductive effects to be revealed.

Using this approach, we found that the E10.5 AoV generates more dHSCs when combined with AoD than on its own (Fig. 2b, compare two rightmost columns). One day later, E11.5 AoD had no positive influence on dHSC generation by AoV (Fig. 2c, compare two rightmost columns). Conversely, the E11.5 AoD produced more HSCs when reaggregated with the AoV than on its own (Fig. 2c, compare two leftmost columns). This inductive effect of AoV on AoD was not observed at E10.5 (Fig. 2b, compare two leftmost columns). These *ex vivo* modelling experiments revealed reciprocal stage-specific effects of AoV and AoD on HSC development, which could be explained by the differential release of factors by the two regions and/or by differences in the competency of the target cells to respond to signals.

### UGRs enhance HSC development in the dorsal aorta

UGRs do not develop HSCs within their own microenvironment (Fig. 1a,b). We tested whether the competent environment of the dorsal aorta can induce dHSCs in UGRs. Chimeric WT//GFP co-aggregates of E11.5 aortas and UGRs were cultured in the presence of 3GF, and then transplanted into irradiated mice (Fig. 2a). Mice injected with these reaggregates could be repopulated with dHSCs coming from the aortas and/or UGRs. The presence of GFP allowed the contributions of aortas and UGRs to the total repopulation level in the recipient mice to be assessed and these are presented in the two separate columns (Fig. 2d). Long-term haematopoietic repopulation originated exclusively from the dorsal aorta and not from UGRs (Fig. 2d), demonstrating that the dorsal aorta cannot induce dHSC generation in the UGRs. We then investigated whether UGRs could induce dHSCs in the dorsal aorta. When comparing aorta alone to aorta cultured with UGRs, we found that the presence of UGRs significantly enhanced HSC development in the dorsal aorta (Fig. 2e). This positive effect of UGRs was observed for both AoD and AoV at E10.5 and E11.5 (Supplementary Fig. 3).

### SCF expression is involved in polarized HSC development

**Figure 3:**
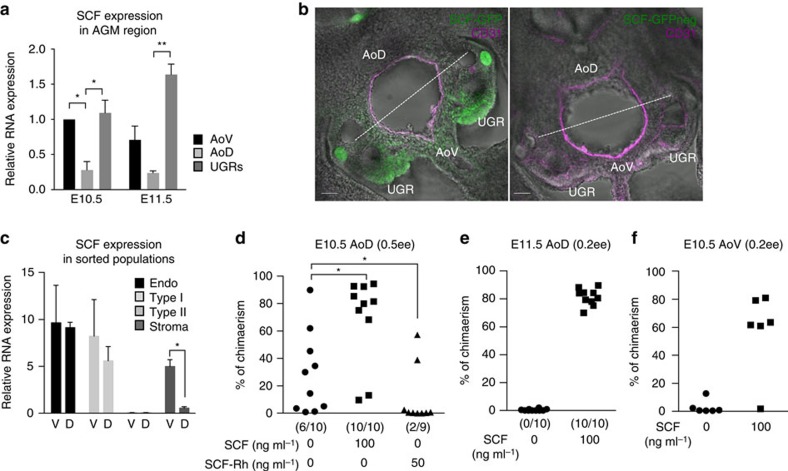
Involvement of polarized stem cell factor in HSC development. (**a**) qRT–PCR on fresh AoV, AoD and UGRs at E10.5 and E11.5 showed high expression levels of stem cell factor (SCF) in AoV and UGRs, compared with AoD (data are mean±s.e.m; *P<0.05, **P<0.01, *t*-test; three independent experiments). No significant difference was observed between E10.5 and E11.5 expression level in any of the tissues. (**b**) Expression of SCF-GFP and CD31 determined by immunostaining on thick section (300 μm) of SCF-GFP-positive E10.5 AGM region and on SCF-GFP-negative control. Bars, 50 μm. (**c**) Expression of SCF in sorted populations from fresh E10.5–E11.5 AoV (V) and AoD (D) determined by qRT–PCR. Endo, endothelial population (VC^+^CD45^−^CD43^−^); type I, pre-HSCs type I (VC^+^CD45^−^CD43^+^); type II, pre-HSCs type II (VC^+^CD45^+^); stroma, stromal population (VC^−^CD45^−^CD43^−^). (*P<0.05, *t*-test; five independent experiments). (**d**) E10.5 AoD were cultured as reaggregates in the presence of Il3 and Flt3L with or without SCF and human SCF antagonist (SCF-Rh). (0.5 e.e. per recipient; three independent experiments). (**e**,**f**) E11.5 AoD (two independent experiments) (**e**) and E10.5 AoV (two independent experiments) (**f**) were cultured as explants with or without SCF (no other cytokines); (0.2 e.e. per recipient).

The results described above could be explained by the differential production of factors by the AoD, AoV and UGRs. We have recently shown that SCF is a potent inducer of early HSC precursors in an *ex vivo* system^17^. Quantitative reverse transcription-PCR (qRT–PCR) showed that SCF expression in the AGM region is polarized, exhibiting higher levels in AoV and UGRs compared with the AoD (Fig. 3a). Clear segregation between the dorsal and ventral compartments of the AGM region can be readily visualized by enhanced expression of SCF in the AoV and UGRs using SCF-GFP knock-in mice^32^ (Fig. 3b) or direct detection of SCF protein (Supplementary Fig. 4). Both non-endothelial stroma and endothelial cells expressed SCF at high levels, but the former showed clear dorso–ventral polarization in SCF expression (Fig. 3c). Importantly, we found that the pre-HSC type I population also expressed SCF, suggesting that a positive-autocrine loop may be involved in their development (Fig. 3c).

We then tested whether SCF can induce dHSC formation in the AoD. Addition of soluble SCF efficiently induced dHSC generation from E10.5 and E11.5 AoD explants (Fig. 3d,e). SCF could also enhance dHSC generation by AoV (Fig. 3f). Conversely, recombinant soluble SCF receptor (SCF-Rh), which binds SCF with high affinity and blocks activation of the cKit receptor, significantly reduced the number of repopulated mice transplanted with AoD cultures compared with SCF-treated or untreated control explants (Fig. 3d).

### Shh signalling enhances dHSC generation

**Figure 4:**
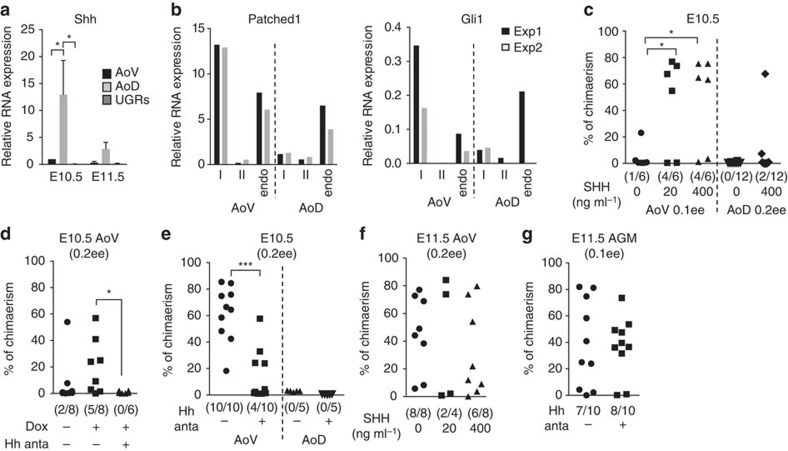
Sonic Hedgehog is a positive modulator of pre-HSC type I. (**a**) Expression level of Sonic Hedgehog (Shh) in E10.5 and E11.5 AGM region determined by qRT–PCR. (data are mean±s.e.m; *P<0.05, *t*-test; E10.5: three independent experiments and E11.5: two independent experiments). (**b**) Patched1 and Gli1 expression in endothelial cells (endo: VC^+^CD45^−^CD43^−^), pre-HSCs type I (I: VC^+^CD45^−^CD43^+^) and type II (II: VC^+^CD45^+^) sorted from E11.5 AoV and AoD (two independent experiments). (**c**) E10.5 AoV and AoD explants were cultured in presence of Shh recombinant protein before transplantation (AoV: 0.1 e.e. per recipient; two independent experiments and AoD: 0.2 e.e. per recipient; three independent experiments). (**d**) E10.5 AoV and doxycyline-inducible OP9-Shh were co-aggregated and cultured in presence or absence of doxycycline and/or Hedgehog (Hh) antagonist (200 nM) before transplantation (0.2 e.e. per recipient; two independent experiments). (**e**) 10.5 AoV and AoD co-aggregated with OP9 were cultured in presence of three growth factors with Hh antagonist before transplantation; (0.2 e.e. per recipient; two independent experiments). (**f**) E11.5 AoV explants were cultured in presence of Shh recombinant protein before transplantation; (0.2 e.e. per recipient; two independent experiments). (**g**): E11.5 AGM reaggregates were cultured in presence of Hh antagonist before transplantation; (0.1 e.e. per recipient; two independent experiments). (**c**,**d**,**f**,**g**) In all these experiments, tissues were cultured without cytokines. Hh anta, Hh antagonist; Dox, doxycycline.

Since dissected AoD tissue includes the notochord, it is likely to be a rich source of Shh^47^. We confirmed this by qRT–PCR (Fig. 4a) and by analysis of GBS (Gli-binding site)-GFP^48^ reporter mice for Hh pathway activity (Supplementary Fig. 5). A significantly higher level of Hh activity was observed in the AoD compared with the AoV tissue (Supplementary Fig. 5). We therefore tested whether Shh could be responsible for the positive impact of AoD on AoV-derived dHSCs. Indeed, addition of recombinant Shh protein to cultures lacking exogenous cytokines significantly increased HSC development in E10.5 AoV (Fig. 4c). Similarly, a Shh doxycycline-inducible OP9 cell line (doxOP9-Shh; Supplementary Fig. 6) co-aggregated with E10.5 AoV cells, also increased HSC development from AoV after doxycycline induction and this was abrogated in the presence of an Hh antagonist (Fig. 4d). The explant and co-aggregation analyses above suggest that the inductive effect of AoD on AoV observed at E10.5 is at least partly mediated by Shh.

At E11.5, addition of Shh could no longer induce HSC development in AoV (Fig. 4f), which is in line with the more subtle effect of AoD on AoV at this stage (Fig. 2c). In keeping with this result, addition of the Hh antagonist inhibited HSC development in the E10.5, but not in the E11.5 AGM region (Fig. 4e,g). Notably, qRT–PCR analysis revealed significant expression levels of Patched1 (Hh receptor) and Gli1 (Hh target) in pre-HSCs type I, but almost no expression of these genes in pre-HSC type II (Fig. 4b). These data suggest that Shh signalling acts specifically on pre-HSCs type I and, as E10.5 AGM contains almost exclusively pre-HSCs type I (ref. 16), they provide an explanation for the stage-specific effect of Shh.

Why are dHSCs absent in the Shh-rich AoD *in vivo*? We found that expression of Patched1 and Gli1 is considerably lower in pre-HSC type I derived from the AoD compared with AoV (Fig. 4b), suggesting their reduced sensitivity to Shh. Accordingly, the stimulatory effect of Shh on AoD explants was insignificant compared with its effect on AoV explants (Fig. 4c). Low sensitivity of dorsally localized pre-HSC type I to Shh may be responsible for attenuated maturation of HSCs in AoD *in vivo*.

### BMP signalling is downregulated in the dHSC lineage

**Figure 5:**
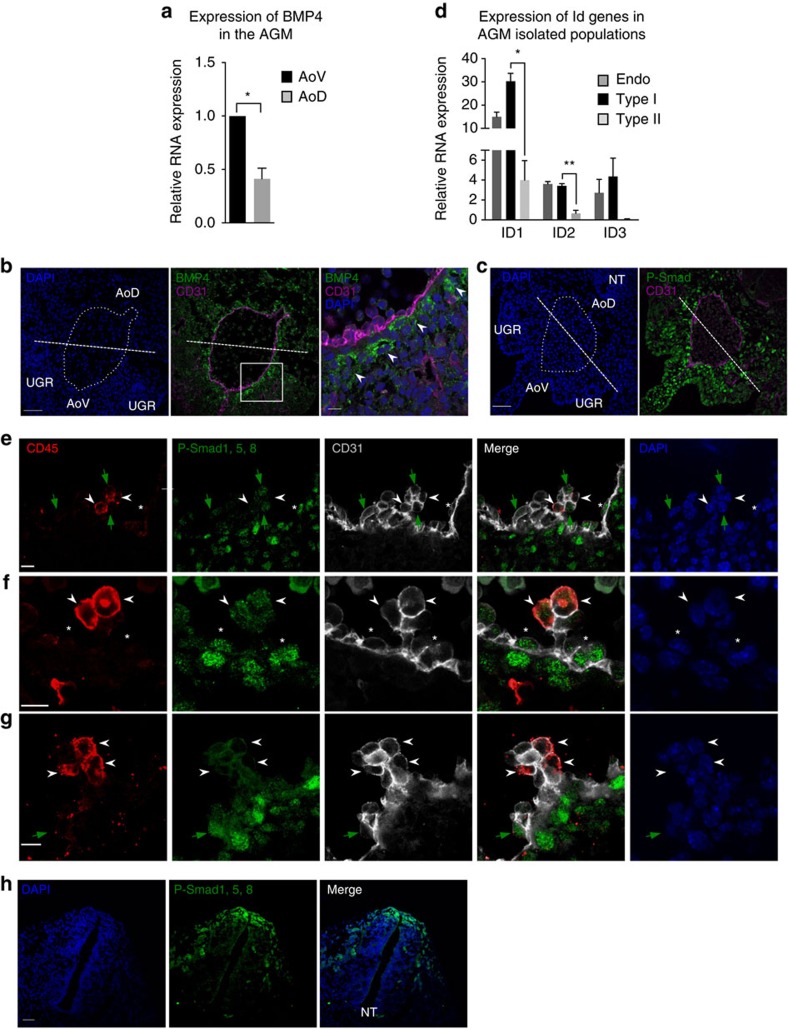
Bone morphogenetic protein signalling is downregulated in dHSC lineage. (**a**) Expression of bone morphogenetic protein 4 (BMP4) at E10.5 determined by qRT–PCR; (data are mean±s.e.m.; *P<0.05, *t*-test; three independent experiments). (**b**) Expression of BMP4 in the E10.5 AGM region determined by immunostaining on frozen sections. Bars, 50 μm. Zoomed image shows the subendothelial localization of BMP4 (arrowheads). Bars, 10 μm. (**c**) Expression of phosphorylated-Smad (P-Smad) in the E10.5 AGM region determined by immunostaining on frozen sections. Bars, 50 μm. (**d**) Id genes expression in endothelial cells, pre-HSCs type I and type II directly isolated from E10.5 and E11.5 AoV determined by qRT–PCR. Endo, endothelial population (VC^+^CD45^−^CD43^−^); type I, pre-HSCs type I (VC^+^CD45^−^CD43^+^); type II, pre-HSCs type II (VC^+^CD45^+^); stroma, stromal population (VC^−^CD45^−^CD43^−^). (Data are mean±s.e.m.; *P<0.05, **P<0.01; *t*-test; five independent experiments). (**e**–**g**) Expression of P-Smad, CD31 and CD45 in the endothelium and haematopoietic clusters of E10.5 dorsal aorta. White arrowheads indicate cells with pre-HSC type II phenotype (CD31^+^CD45^+^); green arrows show (CD31^+^CD45^−/low^) cells budding out of the dorsal aorta and expressing P-Smad; asterisks indicate CD31^+^CD45^−^ cells expressing P-Smad within the endothelium. Bars, 10 μm. A positive control showing P-Smad staining in the dorsal part of the neural tube can be found in **h**.

The emergence of dHSCs in the AoV has been previously attributed to ventrally localized BMP signalling 34,36,49. As expected, qRT–PCR showed that BMP4 is expressed at higher levels in the AoV compared with the AoD (Fig. 5a). Accordingly, BMP4 and phosphorylated forms of Smad1,5,8 (P-Smad), indicative of active Bmp signalling^50^, were abundant in the ventral subaortic mesenchyme (Fig. 5b,c,e–g; Supplementary Fig. 7).

**Figure 6:**
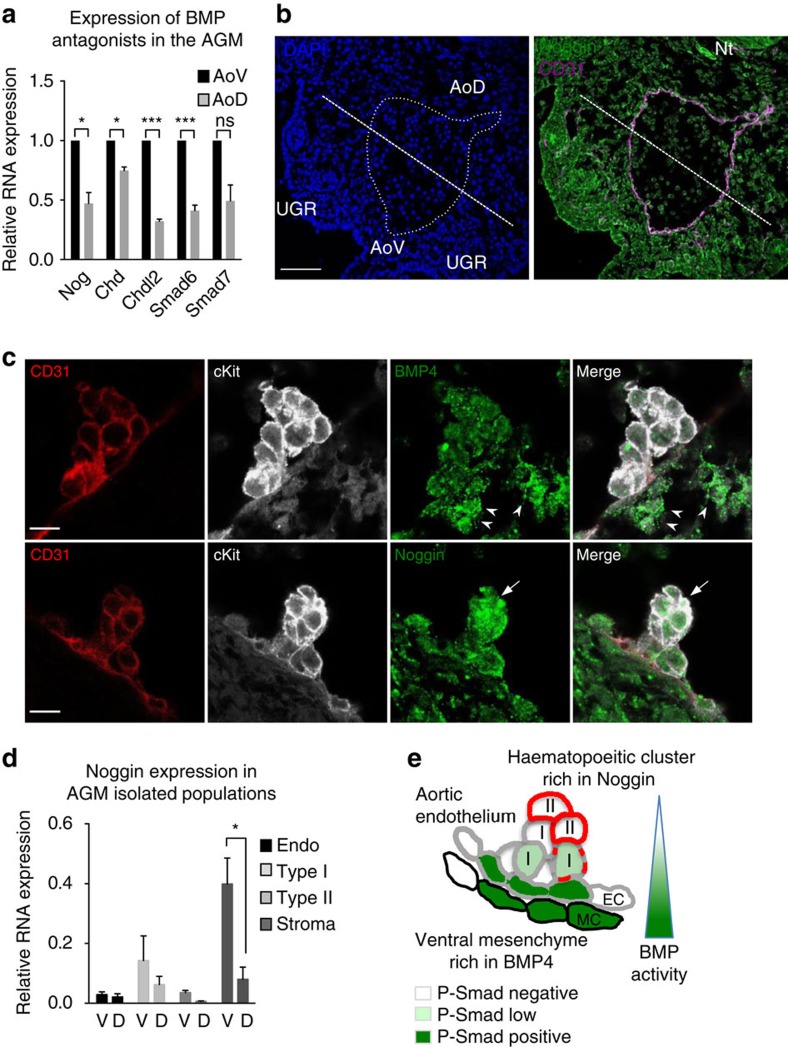
Haematopoietic clusters are exposed to low concentration of BMP4 and high levels of Noggin. (**a**) Expression of BMP antagonists at E10.5 determined by qRT–PCR (data are mean±s.e.m.; *P<0.05, ***P<0.005; *t*-test; three independent experiments). (**b**) Expression of Noggin in the E10.5 AGM region determined by immunostaining on frozen sections. Note the expression of Noggin in the notochord (Nt) as expected. Bar, 50 μm. (**c**) Expression of Noggin and BMP4 in intra-aortic clusters characterized by cKit and CD31 expression. Note that BMP4 is mainly expressed underneath the dorsal aorta (arrowheads), while Noggin is expressed in the cluster (arrows). Bars, 10 μm. (**d**) Expression of Noggin in isolated populations from E10.5 and E11.5 AoV (V) and AoD (D) determined by qRT–PCR. (*P<0.05, *t*-test; five independent experiments). (**e**) Model showing downregulation of BMP activity in dHSC lineage. BMP4 is mainly expressed in the ventral mesenchyme, while Noggin is found in haematopoeitic clusters. Accordingly, BMP activity, assessed by the phosphorylation of Smad1,5 and 8 (P-Smad), is high in mesenchymal cells underneath the aortic endothelium and in some endothelial cells (CD31^+^CD45^−^) of the aortic endothelium and decreases in the haematopoeitic clusters. While some pre-HSC type I cells (CD31^+^CD45^−/low^) exhibit BMP signalling at a low level, acquisition of CD45 (shown in red) is accompanied by a complete loss of BMP activity. EC, endothelial cells; MC, mesenchymal cells; I, pre-HSC type I; II, pre-HSC type II.

However, when we analysed BMP targets genes (*Id1*, *Id2* and *Id3*) in cell populations directly isolated from the embryo, we found that *Id* genes are highly expressed in endothelial cells and pre-HSCs type I, but their expression is significantly lower in pre-HSC type II (Fig. 5d), suggesting a decrease of BMP activity between pre-HSC type I and type II stage. Accordingly, at the morphological level, P-Smad is highly expressed in some luminal aortic endothelial (CD31^+^CD45^−^) cells and at the base of intra-aortic haematopoietic clusters (Fig. 5e–g; Supplementary Fig. 7). Within the clusters, P-Smad levels were lower in CD31^+^CD45^−/low^ cells (Fig. 5e,g, green arrows) and further reduced in cells localized at the periphery of the cluster that acquired CD45, which, together with CD31, defines the pre-HSC type II phenotype (Fig. 5e–g, white arrowheads; Supplementary Fig. 8). Taken together, our data suggest that BMP activity is downregulated during progression of the HSC lineage *in vivo* (Fig. 6e).

How can high BMP4 levels in the AoV be reconciled with the downregulation of BMP activity in the HSC lineage? qRT–PCR showed that BMP antagonists such as Noggin, Chordin and Chordin-like2 are expressed at high levels in the AoV *in vivo* (Fig. 6a). Indicatively, inhibitory Smad6 and Smad7 are also expressed at higher levels in AoV, compared with AoD (Fig. 6a). Furthermore, immunostaining experiments confirm the preferential expression of Noggin in the ventral domain of the AGM (Fig. 6b). Thus, BMP antagonists may be responsible for downregulation of BMP signalling in developing HSCs.

Confocal analysis showed that BMP4 is mainly expressed in subendothelial mesenchyme and is barely detectable in intra-aortic clusters *in vivo* (Figs 5b and 6c; Supplementary Fig. 9a). By contrast, although Noggin is also expressed in subendothelial mesenchyme (Fig. 6b), its expression is readily observed in haematopoietic clusters (Fig. 6c; Supplementary Fig. 9b). Accordingly, qRT–PCR analysis showed that Noggin is highly expressed in the ventral mesenchyme and at lower but significant levels in pre-HSCs type I (Fig. 6d). Thus, downregulation of BMP signalling in the developing HSC lineage observed *in vivo* can be explained by exposure to high levels of Noggin and a low concentration of BMP4 in intra-aortic clusters (Fig. 6e).

### BMP signalling inhibits HSC development

**Figure 7:**
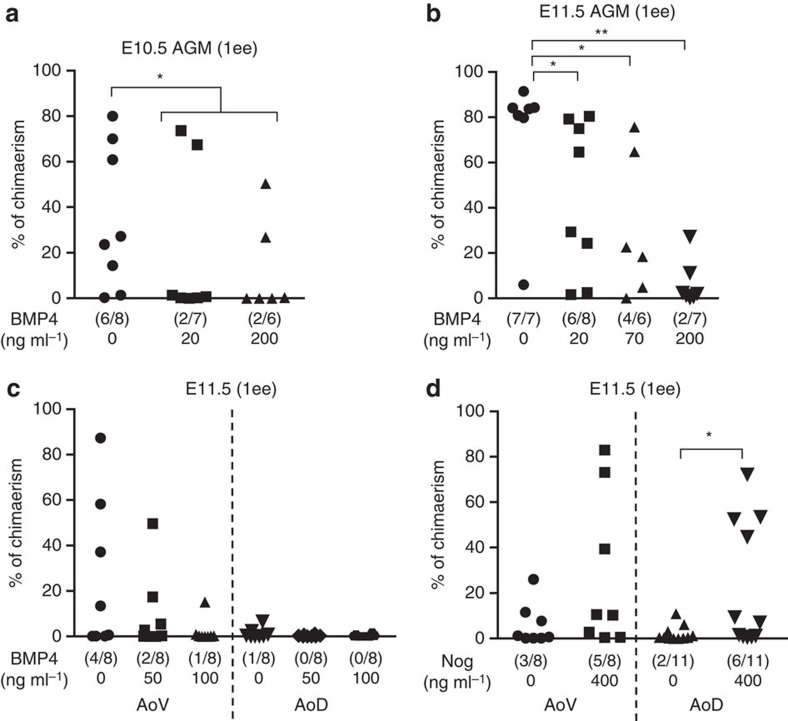
BMP signalling inhibits HSC development in *ex vivo* assays. (**a**) E10.5 AGM reaggregates were cultured with different concentrations of BMP4 in absence of serum and presence of 3GFs before transplantation (1 e.e. per recipient; two independent experiments). (**b**) E11.5 AGM reaggregates were cultured with BMP4 before transplantation (two independent experiments). (**c**,**d**) E11.5 AoV and AoD explants were cultured with BMP4 (two independent experiments) (**c**) or Noggin (two independent experiments) (**d**) before transplantation. (**b**–**d**) In all these experiments, tissues were cultured without serum and without cytokines (1 e.e. per recipient).

To assess the impact of BMP signalling on HSC generation, we used AGM cultures supplemented with BMP4 and assessed dHSC production by the long-term transplantation assay. As the fetal calf serum contains BMP4 (ref. 51), we used a serum-free culture system and demonstrated a consistent dose-dependent inhibitory effect of BMP4 on HSC development in E10.5–E11.5 AGM cultures with concomitant increase in the expression of BMP target genes (Fig. 7a–c; Supplementary Fig. 10a).

By contrast, addition of Noggin (≥100 ng ml^−1^), in the presence or absence of serum, considerably enhanced production of dHSCs in AoV explants and decreased the expression of BMP target genes (Fig. 7d; Supplementary Figs 10b and 11). Notably, Noggin could also induce HSC development in AoD explants in the presence and particularly in the absence of serum (Fig. 7d; Supplementary Fig. 11). These results suggest that efficient HSC development requires that BMP signalling in the AGM region is kept at relatively low levels.

### Interactions between SCF, Shh and BMP signalling pathways

**Figure 8:**
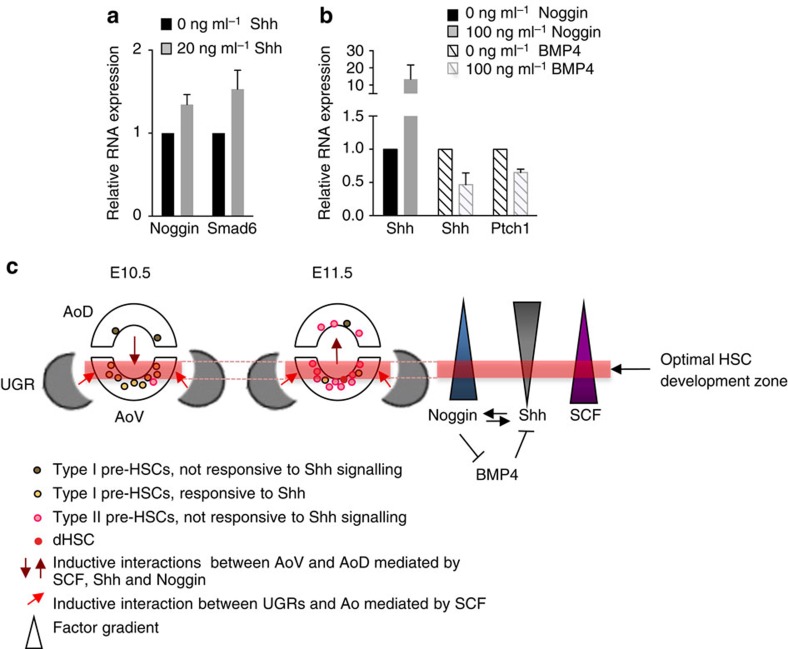
Interplay between SCF, Shh and BMP pathways underpins inductive interactions in the AGM. (**a**) Expression of Noggin and Smad6 in E10.5 AoV explants after treatment with Shh for 24 h assessed by qRT–PCR (data are mean±s.e.m.; three or four independent experiments). (**b**) Expression of Shh and Patched1 (Ptch1) in E10.5 AoV explants after treatment with Noggin (three independent experiments) or BMP4 (three independent experiments) for 24 h assessed by qRT–PCR (data are mean±s.e.m.). (**c**) Model for inductive stage-dependent interactions between AoV and AoD that drive HSC development. Hypothetical effector zone for optimal HSC development in AoV is defined by overlapping gradients of SCF, Shh and BMP antagonists, indicated by a horizontal red bar. Areas adjacent to this zone (for example, AoD) also develop HSCs but with lower efficiency.

The above studies indicate that HSC development in the AGM region is orchestrated in a complex signalling environment consisting of overlapping gradients of SCF, Shh and BMP. We tested whether these signalling pathways can influence each other. We obtained no conclusive data to suggest that addition of Shh/BMP4 modulates SCF expression and vice versa (Supplementary Fig. 12). However, supplementation of AGM cultures with Shh resulted in consistent upregulation of Noggin and Smad6 (Fig. 8a). Noggin in turn enhanced Shh expression, thus creating a positive-feedback loop supporting maturation of HSCs (Fig. 8b). Conversely, BMP4 reduced Shh and Ptch1 expression (Fig. 8b). These results obtained using *ex vivo* modelling system indicate that spatially polarized Shh, SCF and BMP signalling in the AGM region are integral parts of an epistatic regulatory system that drives HSC development in the AGM region *in vivo* (Fig. 8c).

## Discussion

We have shown previously that during murine embryo development definitive HSCs emerge predominantly in the ventral domain of the dorsal aorta (AoV)^6^. This spatially polarized production of HSCs might be explained by different origins of dorsal and ventral endothelium and/or by asymmetric production of key factors involved in HSC development^37^,^52^,^53^ and we reasoned that directional inductive interactions between AGM compartments could be involved. Great insight into inductive interactions in various organs has previously been obtained through *in vitro* modelling^39^. Here we modelled interactions between AGM domains in a co-culture system, which supports HSC development^15^. Using this *ex vivo* system, we demonstrate that at early stages (E10.5) HSC maturation in the AoV region is enhanced by the presence of the AoD. One day later (E11.5), the AoV microenvironment is able to induce HSC development in the AoD, previously thought to be mostly devoid of HSC activity^6^. We also found that UGRs can enhance HSC production from the dorsal aorta, but cannot generate dHSCs themselves, even under influence of the dorsal aorta. Thus, our data strongly suggest that reciprocal stage-specific inductive AoD//AoV interactions and involvement of UGRs are required for execution of the robust development of HSCs *in vivo*.

Our data indicate that previously established dorso–ventrally polarized HSC development^6^ is defined by two main factors. First, our current data show that although the AoD contains pre-HSCs (both type I and type II), their numbers are lower than in AoV, in line with lower intra-aortic cluster formation previously described in mouse AoD^6^,^13^. Second, as shown here, dHSCs can be induced in the AoD by the AoV, and therefore the dHSCs deficiency in AoD cannot be explained solely by asymmetric pre-HSC distribution, but may also be influenced by differences in the microenvironment.

To study this, we focused on SCF, Shh and BMP4, whose expressions are dorso–ventrally polarized in the AGM region^36^,^47^,^49^ (and current data). We found that SCF is an inductive signal that is expressed at high levels in the AoV and UGRs, and can stimulate HSC development in isolated AoD, a region which had previously been considered to be mostly devoid of HSC activity. This is in agreement with a key role of SCF in HSC maturation^17^. We found that the aortic endothelial compartment expresses high levels of SCF, suggesting its important role in HSC development comparable to the bone marrow microenvironment of adult HSCs^32^. Importantly, we found that the pre-HSC type I population expresses SCF suggesting a positive-autocrine loop, which could promote HSC development.

Shh signalling in zebrafish is required for aortic angioblast migration and subsequent arterial specification of the dorsal aorta^34^,^54^. We found that in mouse Shh stimulates and a Hh antagonist inhibits the development of HSCs at E10.5 but not at E11.5, in keeping with a previous study^37^. The induction of dHSCs in AoV by AoD is also limited to the E10.5 stage. Since Shh is secreted by the notochord (which is included in AoD-dissected tissue), this stage specificity is likely defined by the predominant presence of pre-HSCs type I at E10.5, which express higher levels of Shh signalling components (Ptch1 and Gli1) compared with pre-HSCs type II. By E11.5, the pre-HSC population is mainly represented by type II cells^15^. Stage-specific loss of sensitivity to Hh signalling was also described in the developing neural tube^55^. Notably, the poor ability of AoD to develop HSCs despite abundant presence of Shh can also be explained by lower levels of Ptch1 and Gli1 detected in AoD- compared with AoV-derived pre-HSC type I. Our *ex vivo* modelling data indicate that AoD-derived Shh is an active inducer of HSC development in the AGM region. This conclusion does not exclude the possibility that Shh secreted by the gut could also reach the dorsal aorta^37^, although by E10.5 these sites are separated by an extended mesentery.

BMP4 signalling is a key factor involved during differentiation of ventral mesoderm and its further specification into haematopoietic cells. In zebrafish, BMP signalling is clearly required during the patterning of the dorsal aorta and for the emergence of dHSCs in the ventral wall^34^. Its role in mouse is less clear due to the early lethality of BMP mutants^56^. Several lines of evidence point to BMP4 as a good candidate regulating HSC development. Indeed, BMP4 is highly expressed in the ventral mesenchyme underneath the dorsal aorta^34^,^36^,^49^; some reports suggested its role in controlling dHSC emergence^36^,^57^,^58^. However, the *in vitro* systems used likely assayed the maintenance of dHSCs, rather than their maturation. It was also reported that BMP4 signalling can define their differentiation potential^59^. BMP4 is also involved in the regulation of essential haematopoietic transcription factors such as Scl/Gata2/Fli1 and Runx1 (refs 60, 61). Here we analysed BMP signalling activity in the dHSC lineage in the AGM region. We show that *in vivo* the pre-HSC type I to type II transition is accompanied by a downregulation of BMP targets (Id genes). This correlates with our data demonstrating that BMP activity is downregulated in intra-aortic clusters and the observations of others that Runx1 expression is attenuating in the developing HSC lineage^60^,^62^,^63^. How is this decrease of BMP activity achieved *in vivo*, despite the presence of BMP4 in AoV? It has previously been noted that in amphibian embryos several BMP inhibitors are also expressed in AoV^34^. Similarly, our analysis of the embryo showed high expression of a number of BMP antagonists as well as inhibitory Smad6 and Smad7 in mouse AoV that may counteract BMP4 action in HSC lineage. Furthermore, we found that in the AGM region BMP4 and Noggin are spatially segregated: Noggin being present in haematopoietic clusters and BMP4 being mainly expressed underneath the aortic endothelium. Therefore, maturing HSCs in clusters are exposed to low BMP4 concentration and high concentration of the BMP antagonist Noggin. Furthermore, our qRT–PCR analysis shows that the pre-HSC type I population expresses Noggin, which possibly creates a very effective shield that protects them from BMP4. Accordingly, our *ex vivo* analysis strongly suggests that downregulation of BMP signalling is functionally important for HSC development in the embryo. Indeed, forced BMP signalling activation by the addition of BMP4, strongly inhibits HSC development, and conversely the addition of Noggin stimulates HSC development in E10.5–E11.5 AGM cultures. These results are in line with recent observation that deletion of Smad4, a common transducer for BMP4/TGFβ signalling, markedly augments the formation of intra-aortic clusters^64^. Our data do not exclude the possibility that BMP4 is essential for specification of mouse dHSCs at earlier stages, as described in the zebrafish model, where BMP signalling is required for HSC development at stages closer to mouse E8.5 (ref. 34).

Our analysis indicates that all three signalling pathways studied can cooperate for HSC development (Fig. 8c). Notably, the interplay of Shh and BMP pathways is broadly involved in development. For example, counter gradients of polarized Shh and BMP signalling in the developing spinal cord specify neuronal subsets along the dorso–ventral axis^65^, and the dorsal aorta resembles the neural tube with inverse orientation of Shh- and BMP-secreting centres^34^. However, we detected an antagonistic relationship between Shh and BMP pathways. At the molecular level, Shh can induce Noggin and Smad6 expression, thus inhibiting BMP4 signalling. In turn, BMP4 suppresses and, accordingly, Noggin enhances Shh signalling. Cooperation between Shh and Noggin has been previously described as critically important for developmental specification of somitic, neural and hair follicle cells^66^,^67^,^68^. Our *in vitro* data suggest that the feed-forward loop Shh→Noggin/Noggin→Shh is also involved in HSC development *in vivo*.

We propose a model where the polarized secreted factors form complex fields of gradients *in vivo*, which define an effector zone for optimal HSC development in the dorsal aorta and lead to the ventrally shifted appearance of dHSCs (Fig. 8c). Of interest, intra-aortic clusters are abundant in ventro–lateral positions^69^, which may reflect the position of this zone. The dissection close to such a zone could lead to accidental inclusion of powerful dHSCs in AoD samples observed here. Furthermore, it is possible that spatial segregation of co-operating and spatial overlap of antagonizing factors may also be important for adjustment of HSC development *in vivo*. Indeed, although the pool of pre-HSCs in the AGM region markedly expands during E9.5–11.5 (Rybtsov *et al.*, submitted), complete maturation of the HSC pool is limited: while the majority of cells reach the pre-HSC type II stage, only one or two dHSCs are generated by the end of E11. Such controlled dynamics of HSC development may be needed to prevent a burst of active haematopoiesis in the AGM region. How exactly HSC maturation dynamics depend on overlapping concentrations of factors requires further analysis. Although *ex vivo* modelling is a powerful tool to dissect mechanisms of HSC development *in vivo*, there will likely be some variation in details. For example, spatial polarization in the developing HSC niche may define kinetics of HSC development *in vivo.* While we have demonstrated spatial polarization *in vivo* of the factors driving HSC development in our model system, it is currently unclear whether any factors become expressed in a polarized manner within the reaggregates and as such, whether polarization is also a pre-requisite for HSC maturation. Alternatively, if polarization is not required, the entire reaggregate may replicate the optimal zone for HSC development, resulting in massive generation of dHSCs. The distinction between these two scenarios will require further investigation.

In summary, our *ex vivo* modelling experiments suggest that HSC development in the embryo involves stage-dependent interactions between dorsal, ventral and lateral domains of the AGM region, mediated at least partly by the interplay of SCF, Shh, BMP4 and Noggin. Further detailed analysis will be required to better understand the complexity of the AGM signalling landscape in which HSC development takes place. Such knowledge may lead to development of novel protocols for the generation of definitive HSCs *in vitro* for clinical applications.

## Methods

### Mice

Mice were housed and bred in animal facilities at the University of Edinburgh in compliance with the Home Office regulations. Embryos for experiments were obtained from C57BL/6 CD45.2/2, C57BL/6 GFP mice^46^, SCF-GFP mice^32^ (gift from Sean J. Morrison) and GBS-GFP mice^48^ (gift from James Briscoe). The day of discovery of the vaginal plug was designated as day 0.5. The embryos were additionally staged based on somite pair numbers (E10.5=35–38 sp, E11.5=41–45 sp). C57BL/6 CD45.1/2 females or males were used as recipient mice and C57BL/6 CD45.1/1 as a source of carrier cells. All experiments with animals were approved under a project license 60/3916 granted by the Home Office (UK), University of Edinburgh Ethical Review Committee, and conducted in accordance with local guidelines.

### Explant and reaggregate cultures

Isolated E10.5 and E11.5 AGM regions were subdissected into AoV, AoD and UGRs. The notochord was included in the AoD. AGM regions were either cultured as explants or dissociated by collagenase/dispase and then either self-reaggregated or co-aggregated with OP9 stromal cells. For self-reaggregation, AGM cell suspensions were centrifuged at 430*g* for 12 mn in 200-μl pipette tips sealed with parafilm to form a pullet. For co-aggregation with OP9, cell suspension of 1 e.e. of AGM cells or 1 e.e. of sorted cells were mixed with 10^5^ OP9 cells prior centrifugation. Cell aggregates or explants were cultured at the liquid–gas interface on 0.8-μm nitrocellulose filters (Millipore) for 5 days (37 °C, 5% CO_2_) in 5 ml of IMDM (Invitrogen), 20% fetal calf serum supplemented with L-glutamine (4 mM), penicillin/streptomycin (50 U ml^−1^), and 100 ng ml^−1^ SCF, 100 ng ml^−1^ IL3 and 100 ng ml^−1^ Flt3l (termed 3GF), unless otherwise indicated (all purchased from Peprotech). For generation of chimeric reaggregate cultures, AGM regions isolated from age-matched BL/6 WT and BL/6 GFP embryos were subdissected into AoV, AoD and UGRs, and after cell dissociation combined in reaggreates.

The different culture systems used in our study are listed in Supplementary Table 1, as well as indications as to their efficiency in terms of HSC development.

Mouse recombinant proteins Bmp4, Noggin, Shh and human SCF receptor were purchased from R&D Systems and the Hh antagonist was a gift from Genentech (Dr Stephen Gould).

### Transplantation assay

AGM tissues from C57BL/6 CD45.2/2 embryos were pooled (unless otherwise stated) and cell suspensions obtained after dissociation with collagenase/dispase (Roche) for 40 min at 37 °C. Donor cells were injected intravenously into C57BL/6 CD45.1/2 sublethally irradiated (1,150 rad) mice along with 20,000 C57BL/6 CD45.1/1 bone marrow carrier cells. The amount of transplanted cells is expressed in embryo equivalents, defined as a unit of cells equivalent to the number present in one organ. (for example, 0.2 e.e. corresponds to 20% of cells present in one AGM region). The number of embryo equivalents injected for each experiment was chosen based on the expected outcome of dHSC numbers, which can vary for a given tissue depending on culture conditions. For example, the use of different culture systems (co-aggregation with OP9 cells, self-reaggregation or explants) in the presence or absence of cytokines gives different HSC outcomes. Depending on the experimental question, we had to adjust the number of embryo equivalents injected to detect a small number of dHSCs (high dose of cells injected) or to avoid saturation of repopulation to allow assessment of inductive factors/tissues (low dose of cells injected).

Long-term haematopoietic repopulation was assessed in peripheral blood between 14 and 16 weeks after transplantation. Peripheral blood was collected by bleeding the tail vein into 500 μl of 5 mM EDTA/PBS, and erythrocytes were depleted using PharM Lyse (BD). Cells were stained with anti-CD16/32 (Fc-block), anti-CD45.1-APC (cloneA20) and anti-CD45.2-PE (clone 104) monoclonal antibodies (eBioscience), and analysed using a FACSCalibur. Data were analysed using the FlowJo software (TreeStar). Recipients of the chimeric reaggregates were analysed for both CD45.2 and aGFP donor markers. Mice exhibiting >5% of donor chimerism were considered to be repopulated with dHSCs. Different groups of repopulated mice were compared using Mann–Whitney *U* statistical tests (*P<0.05; **P<0.01; ***P<0.005).

### Fluorescence-activated cell sorting

Single-cell suspensions from the AGM region were prepared by dispase/collagenase-mediated dissociation. Antibodies used for staining of cells were anti-CD45-BV450 (BD Horizon, clone 30F11, 1:100), anti-CD45-AF700 (1:100, clone 30F11, BD pharmingen), anti-VE-cadherin-AF647 or -AF488 (1:100, Clone eBioBV13, Biolegend) and biotinylated anti-VE-Cadherin (1:50, clone 11.D4.1, Pharmingen), followed by incubation with streptavidin-PE (1:600, BD Pharmingen), anti-CD43-PE (1:200, clone eBioR2/60, eBioscience), anti-cKit-APC (1:100, clone 2B8, eBioscience), anti-CD31-PE (1:200, MEC13.3, Pharmingen), anti-Sca1-V500 (1:100, clone D7, BD Bioscience), anti-CD41-BV421 (1:100, clone MWreg30, Biolegend) and anti-Ter119-PerCp-Cy5.5 (1:100, clone TER119, eBioscience). 7-Aminoactinomycin D viability staining solution was used to exclude dead cells, and gates were set using appropriate fluorescence minus one controls. Sorting was performed on a FACSAriaII using the FACSDiva software.

### Quantitative reverse transcription–PCR

Total RNA was isolated from AoV, AoD, UGRs (using at least 4 e.e. of tissue for each sample) and OP9 with Qiagen RNeasy microkit or minikit (Qiagen), and complementary (cDNA) was prepared using SuperScriptVILO cDNA Synthesis (Invitrogen). The qRT–PCR was performed using the LightCycler 480 SYBR Green I MasterMix (Roche). For gene expression on a small number of cells, total RNA was isolated from 200 cells directly FACS-sorted into a PCR tube containing 10 μl of × 2 Reaction Mix (CellsDirect, Invitrogen) and 0.2 μl RNase inhibitor (SUPERase-In Ambion AM2694). cDNA preamplification of the genes of interest was achieved by adding directly Superscript III/Taq mix (CellsDirect) and primers (10 μM each) to the cell lysate (PCR programme: 50 °C for 15 min; 95 °C for 2 min; 18 cycles of: 95 °C for 15 s; 60 °C for 4 min). A universal probe library strategy with Lightcycler 480 probes mastermix kit (Roche) was used to perform qRT–PCR on these samples. Duplicate reactions were set-up with diluted preamplified cDNA. PCR primers were designed using the Roche software. All experiments were performed in biological duplicates or more. Expression values were normalized against the TATA-binding protein, and standard error of the mean (s.e.m.) were calculated and plotted using the Prism6 software (GraphPad). Primer sequences can be found in Supplementary Table 2. The expression data are expressed as mean±s.e.m. Statistical analysis were performed using Student's *t*-tests. Significance is indicated in the figures with the following convention: (*P<0.05; **P<0.01; ***P<0.005).

### Shh doxycycline-inducible OP9 cell line (doxOP9-Shh)

cDNA coding for Shh was cloned into pPBhCMV1-cHA-IRESVenuspA vector, a doxycycline-inducible bicistronic expression vector—gift from H. Niwa^70^—allowing both Shh and Venus to be expressed upon induction. OP9 cells (*n*=100,000) were electroporated with this plasmid using NEON transfecting system (Invitrogen). The next day, the electroporated cells were cultured in the presence of 1 μg ml^−1^ doxycycline (Clontech). After 24 h of treatment, doxOP9-Shh cells expressing Venus were sorted using FACSAriaII and plated in culture without doxycycline. After 1 week in culture, a second sort was performed to remove any cells constitutively expressing Venus. Before co-aggregation with AGM cells, doxOP9-Shh cells were cultured for 24 h in regular growth medium supplemented with 1 μg ml^−1^ doxycycline. Co-aggregates were cultured in the presence of doxycycline for 5 days. OP9 cells were grown at 37 °C in a humidified atmosphere (5% CO_2_). The growth medium was IMDM (Invitrogen) supplemented with 20% fetal calf serum and penicillin/streptomycin (50 U ml^−1^).

### Immunofluorescence

Embryos were fixed in 4% paraformaldehyde at 4 °C overnight (P-Smad staining) or for 45 mn (BMP4 and Noggin stainings) and embedded in OCT by flash freezing on dry ice. Transverse sections (7 μM) were produced using a CM1900 Crystat (Leica). Sections were permeabilized in PBS/0.1% Triton/1% BSA for 1 h, before blocking for 30 mn in PBS/10% fetal calf heat-inactivated serum. Immunostainings were performed using rabbit anti-P-Smad1,5,8 (1:100, clone D5B10, Cell Signaling Technology), goat anti-BMP4 (1:50, clone N-16, Santa Cruz Biotechnology), goat anti-Noggin (1:100, clone AF 719, R&D systems), rabbit anti-SCF (1:50, ab64677, Abcam), unconjugated rat anti-CD31 or PE-conjugated anti-CD31 (1:50, MEC13.3, Pharmingen), goat anti-CD45 (1:100, clone AF 114, R&D systems) and rat anti-cKit (1:100, clone 2B8, Biolegend), followed by incubation with anti-goat Alexa fluor 488 (1:100, Invitrogen), anti-goat NL577 (1:100, R&D systems), anti-rabbit Alexa fluor 488 (1:100, Invitrogen) and anti-rat Alexa fluor 647 (1:100, Invitrogen). Images were acquired with an inverted confocal microscope (SP8) and processed using FiJi software.

## Additional information

**How to cite this article:** Souilhol, C. *et al.* Inductive interactions mediated by interplay of asymmetric signalling underlie development of adult haematopoietic stem cells. *Nat. Commun.* 7:10784 doi: 10.1038/ncomms10784 (2016).

## Supplementary Material

Supplementary Information Supplementary Figures 1-12, Supplementary Tables 1-2.
