# Supplementary material for: Inductive interactions mediated by interplay of asymmetric signalling underlie development of adult haematopoietic stem cells
Source: Nat Commun. 2016 Mar 8;7:10784. doi: 10.1038/ncomms10784 (PMC4786750; doi:10.1038/ncomms10784)
Supplement: Supplementary Information — Supplementary Figures 1-12, Supplementary Tables 1-2. [file ncomms10784-s1.pdf]

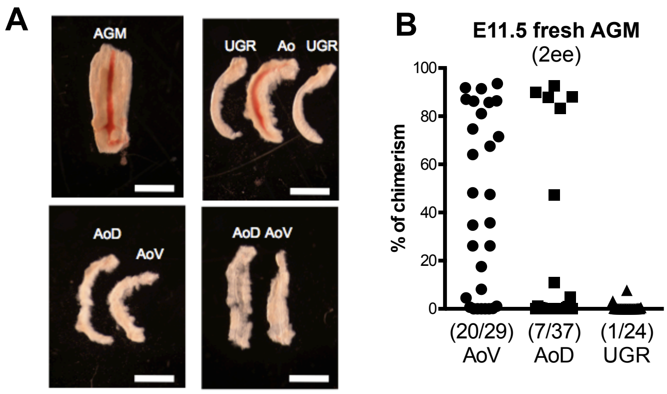

Supplementary Figure 1: Polarized distribution of dHSCs in E11.5 AGM region.
A: Sub-dissection of E11.5 AGM region into UGRs, AoV and AoD. Bars: 1mm.
B: E11.5 AoV, AoD and UGR were injected directly into irradiated mice (2ee/recipient); (three independent experiments).
AGM: Aorta-Gonad-Mesonephros region; Ao: dorsal Aorta; UGRs: Uro-Genital Ridges; AoV: Ventral domain of the dorsal Aorta; AoD: Dorsal domain of the dorsal Aorta.

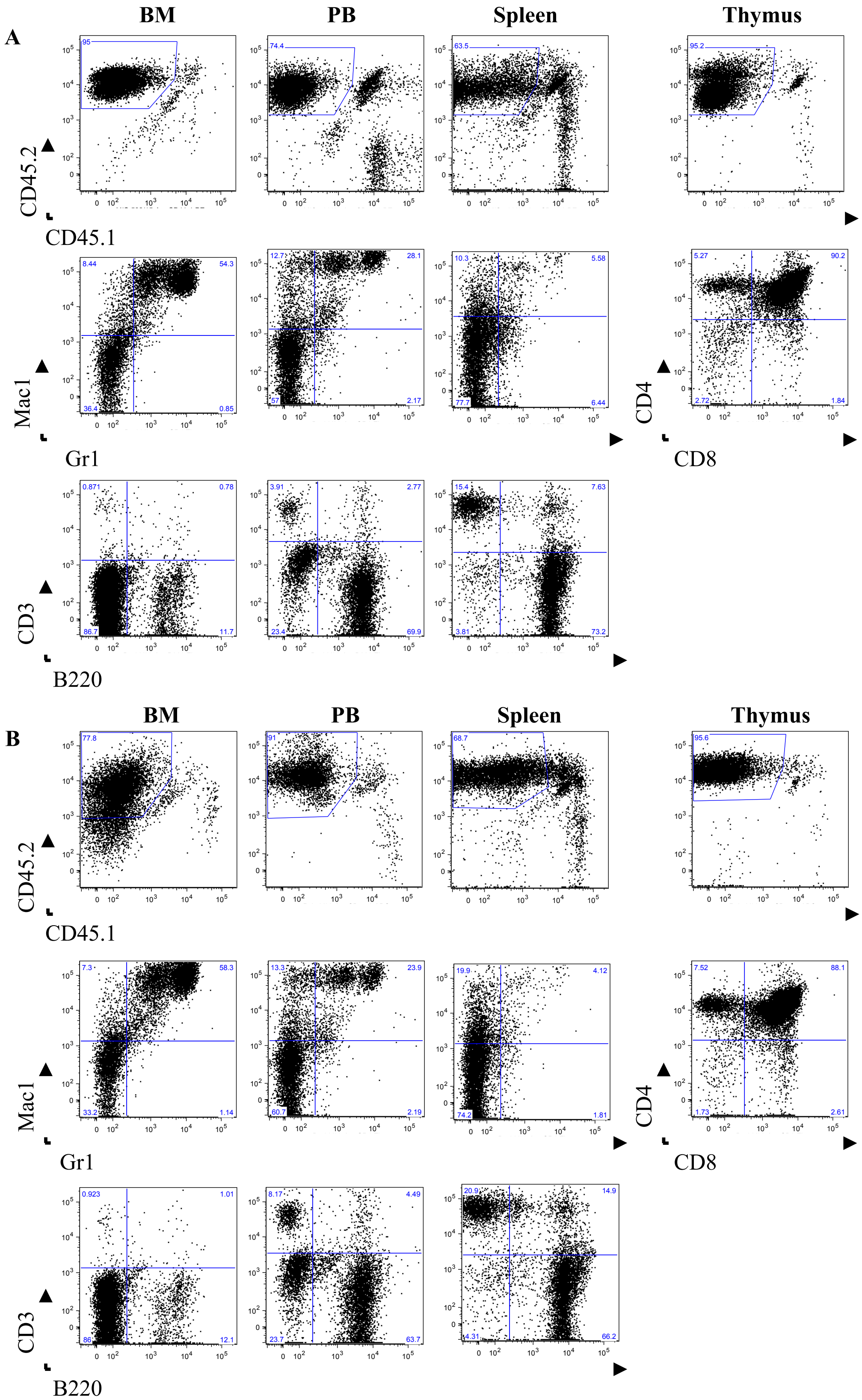

Supplementary Figure 2: Examples of multilineage analysis in engrafted recipient mice that were injected with AoV cells (A) or AoD cells (B).

Dot plots are representative of donor derived (CD45.2+) myeloid (Mac1+ and Gr1+) and lymphoid (CD3+ and B220+) cell populations in bone marrow (BM), peripheral blood (PB), spleen and thymus of recipient mice 6 months after transplantation.

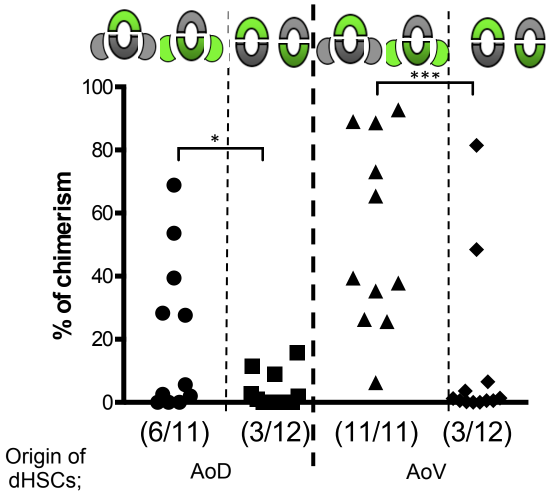

Supplementary Figure 3: The presence of UGRs enhances HSC development in AoD and AoV. E11.5 aortas and UGRs from WT and GFP embryos were used to generate chimeric reaggregates as depicted schematically above plots. The reciprocal combination of WT and GFP tissues was used to generate AoV//AoD//UGRs reaggregates. The tissue source of dHSCs is shown separately in the leftmost (AoD) and rightmost (AoV) columns as indicated below the plot; (0.2ee/recipient); (two independent experiments). AoD: Dorsal domain of the dorsal aorta; AoV: Ventral domain of the dorsal Aorta.

A

B

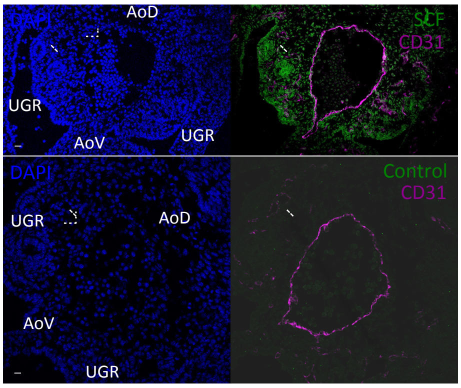

Supplementary Figure 4: **Expression of SCF in E10.5 AGM region.**
A: Expression of SCF and CD31 in E10.5 AGM region determined by immunostaining on frozen section.
B: Immunostaining control with secondary antibody anti-rabbit Al488.
UGRs: Uro-Genital Ridges; AoV: Ventral domain of the dorsal Aorta; AoD: Dorsal domain of the dorsal Aorta; SCF: Stem Cell Factor. Bars: 50μm.

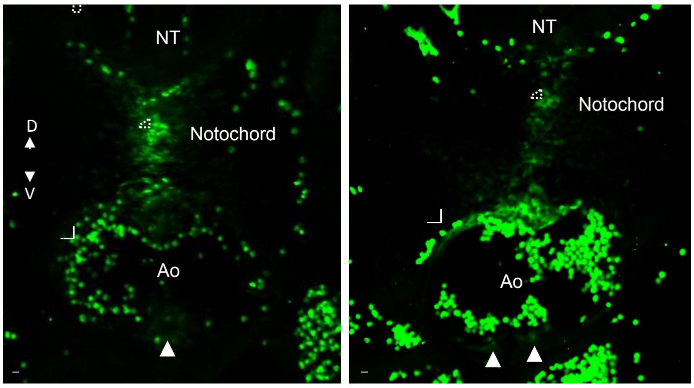

Supplementary Figure 5: **Expression of GBS-GFP in E10.5 AGM region.**
GFP expression on transversal frozen sections of E10.5 GBS-GFP$^+$ AGM region. Arrowheads indicate low GFP expression underneath the aortic endothelium. Bar: 20µm. GBS: Gli Binding Sites; NT: neural tube; Ao: Aorta

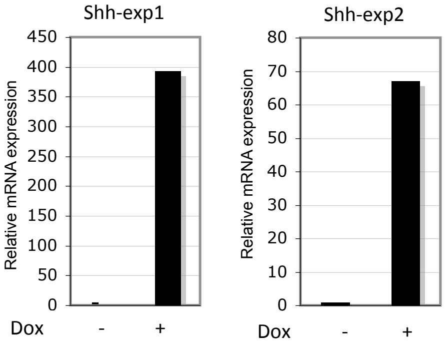

Supplementary Figure 6: **Validation of the doxOP9-Shh cell line.**
Expression of Shh by genetically modified OP9 (doxOP9-Shh) after 24h treatment with doxycycline; (n=2).
Dox: Doxycycline; exp: experiment.

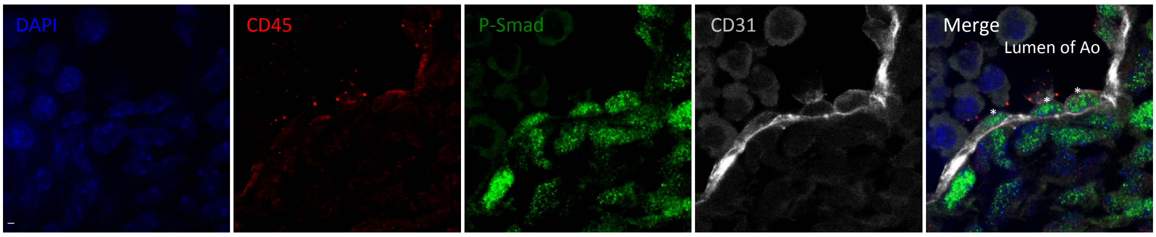

Supplementary Figure 7: Expression of P-Smad in AGM region.
Immunostaining for P-Smad, CD31, CD45 and DAPI showing expressing of P-Smad in endothelial cells ($CD31^{+}CD45^{-}$ cells marked by asterisks) and the underlying ventral mesenchyme. Bar: 10μm.
P-Smad: Phosphorylated form of Smad1, 5, 8; Ao: Aorta.

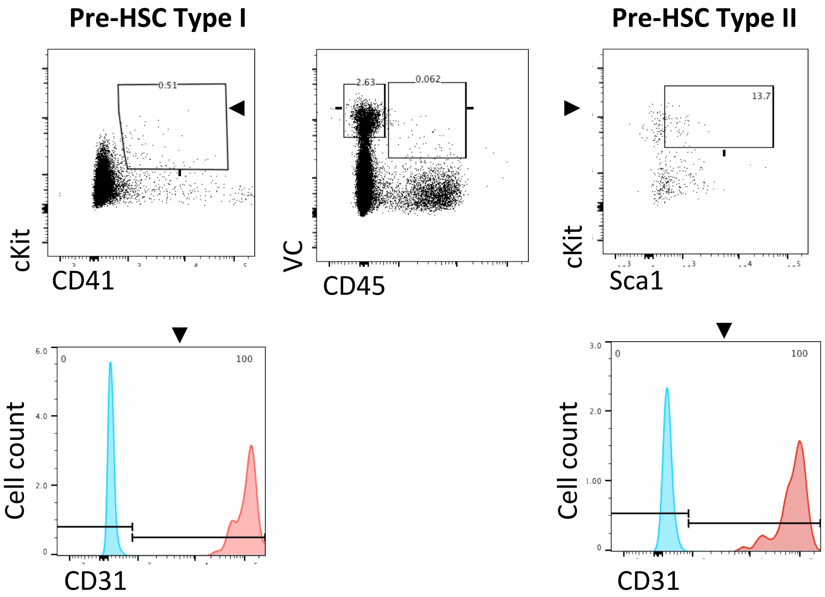

Supplementary Figure 8: CD31 is expressed in Pre-HSCs Type I and Type II. Gating strategy showing the expression of CD31 (red histogram) in pre-HSCs Type I (defined by VC, CD41 and cKit expression) and pre-HSCs Type II (defined by VC, CD45, cKit and Sca1 expression). 100% of pre-HSCs highly express CD31. Blue histogram: isotype control for CD31.

VC: Vascular-endothelial Cadherin

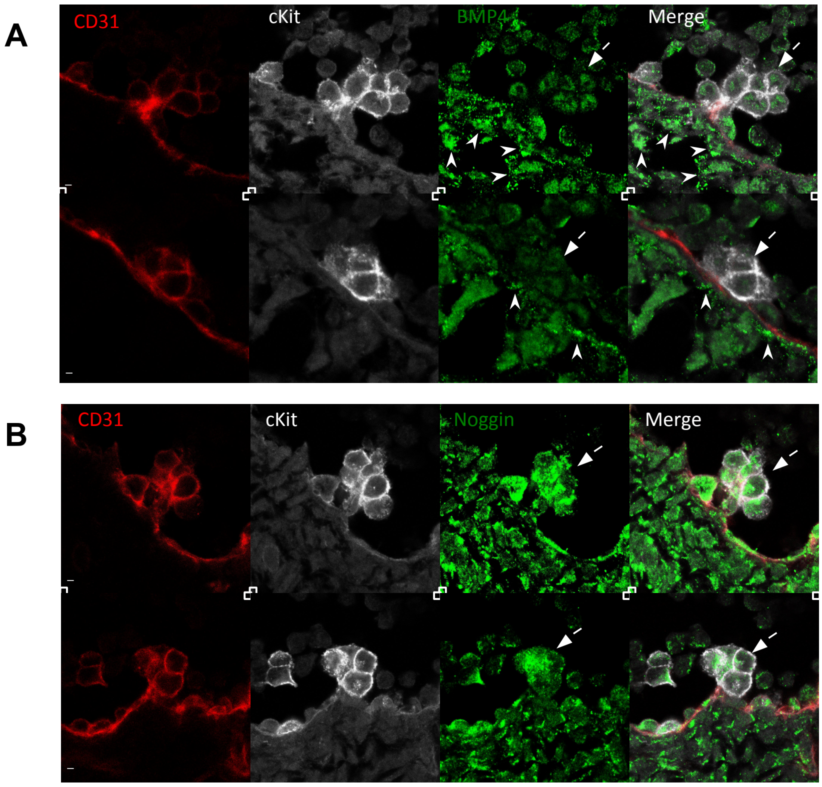

Supplementary Figure 9: BMP4 and Noggin expression in the intra-aortic clusters.
Intra-aortic clusters are characterized by co-expression of cKit and CD31.
A: BMP4 is mainly expressed underneath the aortic endothelium (arrowheads) and barely detected in the clusters (arrows). Bars: 10μm.
B: Noggin is present in the hematopoietic clusters (arrows). Bars: 10μm.
BMP4: Bone Morphogenetic Protein 4.

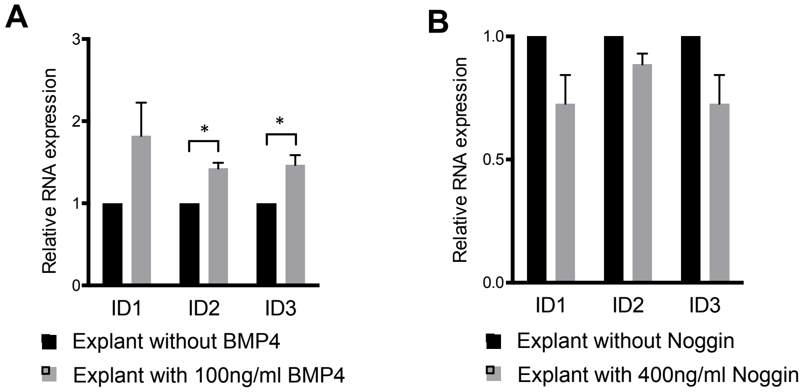

Supplementary Figure 10: Expression of Id genes after addition of BMP4 or Noggin to E11.5 AGM region.

Expression of BMP target genes Id1, Id2, Id3 in E11.5 AGM region after treatment with **(A)** 100ng/ml BMP4 for 24h (three independent experiments) or **(B)** 400ng/ml Noggin for 3 days (three independent experiments). (*$p<0.05$, *t*-test); (data are mean±s.e.m).

BMP4: Bone Morphogenetic Protein 4.

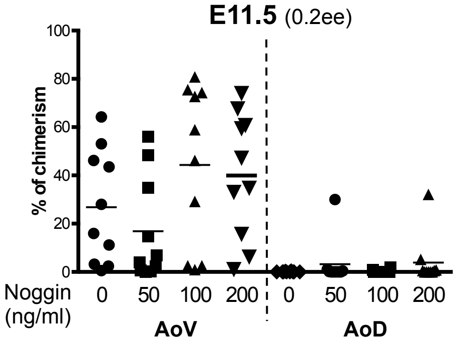

Supplementary Figure 11: Effect of Noggin on HSC development in presence of serum.

E11.5 AoV and AoD explants were cultured for 5 days with Noggin in presence of serum without cytokines and injected separately into irradiated mice; (0.2ee/recipient); (two independent experiments).

AoV: Ventral domain of the dorsal Aorta; AoD: Dorsal domain of the dorsal Aorta.

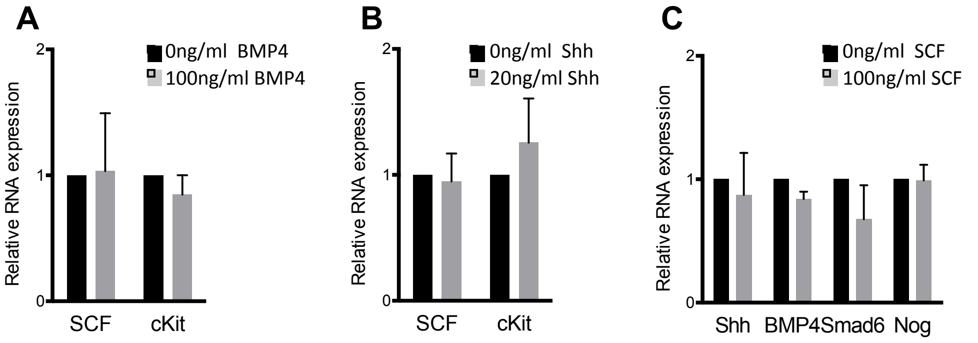

Supplementary Figure 12: Effect of SCF on BMP4 and Shh and vice versa.

A: Expression of SCF and cKit in E11.5 AoV after treatment with BMP4 for 24h; (data are mean±s.e.m.); (three independent experiments).

B: Expression of SCF and cKit in E10.5 AoV after treatment with Shh for 24h; (data are mean±s.e.m.); (two independent experiments).

C: Expression of Shh, BMP4, Smad6 and Noggin in E10.5 AoV after treatment with SCF for 24h; (data are mean±s.e.m.); (three independent experiments).

SCF: Stem Cell Factor; Shh: Sonic Hedgehog; BMP4: Bone Morphogenetic Protein 4.

Supplementary Table1

Summary of the culture systems used in the present study.

Culture systems (ranked from the most to the least efficient)	E10.5		E11.5	
	AoV	AoD	AoV	AoD
Reaggregation with OP9 + cytokines	10/10 (fig. 4E)	0/5 (fig. 4E)	ND	ND
Reaggregation without OP9 + cytokines	5/12 (fig. 2B)	1/12 (fig.2B)	16/20 (fig.2C)	4/20 (fig.2C)
Reaggregation with OP9 – cytokines	2/8 (fig.4D)	ND	ND	ND
Explant -cytokines	1/6 (fig.3F)	0/12 (fig.4C)	7/10 (fig.S11)	0/10 (fig.3E)
Reaggregation without OP9 -cytokine -serum	ND	ND	4/8 * (fig.7C)	1/8 * (fig.7C)

The culture systems are listed in order of their efficiency in terms of HSC development. Representative fraction of repopulated mice obtained after injection of 0.2ee is indicated for each culture condition, for AoV and AoD at E10.5 and E11.5.
(*): 1ee injected.
AoV: Ventral domain of the dorsal Aorta; AoD: Dorsal domain of the dorsal Aorta.ND: Not Done.

Supplementary Table 2

Primers used for qRT-PCR analysis

Name	Sequences	UPL probe
SCF-F	TCAACATTAGGTCCCGAGA	68
SCF-R	ACTGCTACTGCTGTCATTCC	
cKit-F	TCATCTATACGTTGTATGCGTTCAT	25
cKit-R	AAACGAAAGCTTGTGGGAAA	
BMP4-F	GATCTTTACCGGCTCCAGTCT	101
BMP4-R	CCTGGGATGTTCTCCAGATG	
Noggin-F	CGGCCAGCACTATCTACACA	107
Noggin-R	GTTCGATGAGGTCCACCAAG	
Chordin-F	TCTCCACCAGGGACAGCTAC	1
Chordin-R	CAGCACCTCAGCAAAACCT	
Chordin like 2-F	AGAACTTCCGGCTGCTCAC	109
Chordin like 2-R	CTGGGCTGGCTGTAACTTTC	
Smad6-F	GTTGCAACCCCTACCACTTC	70
Smad6-R	GGAGGAGACAGCCGAGAATA	
Smad7-F	ACCCCCATCACCTTAGTCG	63
Smad7-R	GAAAATCCATTGGGTATCTGGA	
ID1-F	TCCTGCAGCATGTAATCGAC	78
ID1-R	GGTCCCGACTTCAGACTCC	
ID2-F	GACAGAACCAGGCGTCCA	89
ID2-R	AGCTCAGAAGGGAATTCAGATG	
ID3-F	CATAGACTACATCCTCGACCTTCA	53
ID3-R	CACAAGTTCCGGAGTGAGC	
Shh-F	CCAATTACAACCCCGACATC	32
Shh-R	GCATTTAACTTGTCTTTGCACCT	
Ptch1-F	TGACAAAGCCGACTACATGC	64
Ptch1-R	GTACTCGATGGGCTCTGCTG	
Gli1-F	CTGACTGTGCCCGAGAGTG	84
Gli1-R	CGCTGCTGCAAGAGGACT	
